# Comparative Biochemical and Transcriptomic Analyses Provide New Insights into Phytoplasma Infection Responses in Cucumber

**DOI:** 10.3390/genes13101903

**Published:** 2022-10-19

**Authors:** Xueting Wang, Qiming Hu, Jiaxi Wang, Lina Lou, Xuewen Xu, Xuehao Chen

**Affiliations:** 1 School of Horticulture and Landscape Architecture, Yangzhou University, Yangzhou 225009, China; 2 Jiangsu Key Laboratory for Horticultural Crop Genetic Improvement, Institute of Vegetable Crops, Jiangsu Academy of Agricultural Sciences, Nanjing 210014, China; 3 Joint International Research Laboratory of Agriculture and Agri-Product Safety, Ministry of Education of China, Yangzhou University, Yangzhou 225009, China

**Keywords:** cucumber, flat stem, phytoplasmas, plant hormones, minerals, RNA-seq

## Abstract

Flat stem and witches’ broom phytoplasma-like symptoms in the cucumber inbred line C17 were observed in a greenhouse at Yangzhou University, China for three consecutive planting seasons; these symptoms resulted in a decreased yield. To better understand the cause of these symptoms, 16S rRNA PCR, plant hormones, mineral elements, and RNA-seq profiling were performed using symptomatic and normal stem samples. The results showed that the causal agent was classified as the *Candidatus* phytoplasma asteris strain, a plant pathogenic prokaryote that could not be cultured in vitro. Measurement of plant hormones showed that auxin, salicylic acid, and jasmonic acid contents were significantly increased, whereas that of ethylene’s immediate biosynthetic precursor, 1-aminocyclopropane-1-carboxylic acid, was decreased in the phytoplasma-infected stems compared with the healthy stems. Furthermore, measurement of mineral element composition showed that magnesium, calcium, sodium, iron, and zinc concentrations significantly changed in the phytoplasma-infected cucumber stems compared with the uninfected stems. Comparative RNA-seq identified 253 differentially expressed genes, including 179 upregulated and 74 downregulated genes. Further analyses suggested that genes related to phenylpropanoid biosynthesis, phenylalanine metabolism, and plant hormone signal transduction contributed to phytoplasma infection. Taken together, this study presents the first in-depth assessment of disease symptoms and biochemical content of cucumber stems known to be infected with phytoplasma.

## 1. Introduction

Cucumber (*Cucumis sativus* L.), belonging to the *Cucurbitaceae* family, is one of the most important vegetables because of its rich nutrient profile and versatile uses in culinary, cosmetic, and therapeutic sectors. The total world production of cucumbers in 2020 was 91,258,272 metric tons on 2,261,318 hectares (FAO STAT 2020, http://faostat.fao.org, accessed on 5 April 2022). Phytoplasmas are biotrophic microbes that lack cell walls. Plants exhibit flat stems, witches’ broom, yellowing, stunting, and lethal yellowing symptoms after infection, which leads to an estimated 30–80% economic loss in agricultural production [1,2,3,4]. Phytoplasmas are transmitted by insects and infect more than 700 plant species worldwide [5]. However, because phytoplasmas are difficult to cultivate in vitro, research on the molecular mechanisms used by plants to defend against phytoplasma infection is limited [6].

High-throughput RNA-sequencing (RNA-seq) has been used to analyze changes in the expression of genes and metabolic pathways involved in plant–phytoplasma interactions [6,7,8]. Genes related to primary and secondary metabolites, the cell wall, hormones, and signaling, as well as diseases-related genes, were reported to be associated with host resistance to phytoplasma infection [7,8]. Photosynthesis-related genes are downregulated in infected grape [9], and both chlorophyll and total soluble sugar production decrease in infected lime [10]. The biosynthesis of some secondary metabolites has positive effects on resistance. Several genes that encode flavonoid biosynthesis, lipid metabolism, and lignin and phenylpropanoid synthesis are upregulated in grape during phytoplasma infection [11,12], and some flavonoid compounds rapidly activate cucumber defense processes against the pathogen [13]. Moreover, cell wall reinforcement-related genes are induced, whereas cell wall degradation genes, which form a primary physical barrier to defend against pathogen invasion, are inhibited in paulownia infected with paulownia witches’ broom (PWB) phytoplasma [7].

In addition, plant hormones including auxin, gibberellin (GA), abscisic acid (ABA), jasmonic acid (JA), salicylic acid (SA), ethylene (ETH), brassinolide (BR), and cytokinin (CTK), play important roles in plant immunity [14]. The indole-3-acetic acid level increase and auxin signals contribute to disease development during plant–pathogen interactions [15]. Isopentenyl diphosphate isomerase and isopentenyl transferase, which are involved in CTK biosynthesis, are induced in infected paulownia and the cytokinin dehydrogenase related to CTK degradation is repressed in coconut leaves infected with phytoplasma, which may result in the CTK concentration increasing during defense responses to pathogen invasion [7,16]. In general, when a plant is infected with phytoplasma, the GA and ABA contents decrease and genes that encode BR synthesis are upregulated [17]. ETH, JA, and SA play crucial roles in plant resistance [18,19]. The JA and SA contents remarkably increase after the grapevine is infected with phytoplasma, and ETH acts in synergy with JA signals to improve plant resistance [18]. The crosstalk among these plant hormones forms part of the plant’s resistance system against pathogen infection. 

Mineral nutrients act as important structural components of plant tissues that influence plant growth and development, and they also affect plant resistance to pathogens by regulating plant morphological structures, growth, and biological processes [20]. Among these mineral nutrients, nitrogen, phosphorus, and potassium are the major elements, and the application of fertilizers containing these elements has crucial effects in fighting against pathogen infections. For example, bacterial leaf spot of tomato decreases after an application of nitrogenous fertilizer, whereas stem rust in wheat increases [21], suggesting that different pathogens vary in their response to nitrogen. Supplying phosphorus and increasing the plant’s potassium concentration enhances resistance in rice and banksia [22,23]. In addition, calcium helps maintain cell membrane stability and increases the activities of resistance-related enzymes, such as superoxide dismutase and peroxidase (POD) [24]. Zinc and iron enhance the ability of plants to defend against pathogens by affecting auxin oxidase activity. Copper harbors unique redox properties and acts as a cofactor for a series of enzymes in response to plant–pathogen interactions [25]. Most mineral elements enhance or inhibit plant resistance against pathogens. However, the mechanisms responsible for these effects in cucumber are unclear.

To date, cucumber infected by phytoplasma has only been reported in Iran with phyllody symptoms [26]. Flat stem and witches’ broom symptoms were observed in the cucumber inbred line C17 growing in a greenhouse at Yangzhou University, China. The symptoms resulted in abortive pollen and decreased cucumber production. To understand the flat stem symptom, nested PCR and microscopic observations were used to determine whether the pathogen was the cucumber flat stem phytoplasma. Furthermore, we analyzed changes in plant hormone levels, mineral nutrient contents, and transcription, in response to the pathogen infection of cucumber, thereby increasing our understanding of cucumber–microbe interactions.

## 2. Materials and Methods

### 2.1. Plant Materials

C17 is a gynoecious inbred line that has a round-shape and good-tasting fruit. The seedlings were cultivated in an experimental greenhouse at Yangzhou University, Yangzhou, China. The organic fertilizer (1200 kg/ha) was applied in soil before transplant, and the compound fertilizer (nitrogen: phosphorus: potassium = 15:15:15%, 45 kg/ha) was supplemented thrice at growth stages of cucumber. The flat stem and witches’ broom symptoms were observed in C17 from the first internode up until plant death, whereas healthy plants exhibited round stems and normal growth. At initial full-bloom stage, the stems from symptomatic and normal plants were collected, with three replications, under the same conditions, for later analyses. 

### 2.2. Detection of Plant Pathogen in Flat Stems

To identify the pathogen in flat stems of infected cucumber, young stems were harvested, fixed in a glutaraldehyde buffer, and observed using light microscopy (Nexcope, Ningbo, China) and transmission electron microscopy (TEM). To further determine whether the pathogen was a phytoplasma, total DNA was extracted using an improved CTAB method [27]. The DNA concentration and quality were evaluated using a Nanodrop spectrophotometer (Thermo Fisher Scientific, Waltham, MA, USA) and agarose gel electrophoresis. The universal primer pairs, P1/P7 and R16F2n/R16R2 for 16S rRNA, were used to detect the existence of phytoplasmas with nested PCR [28,29,30]. The primer sequences are listed in Table S1. PCR products were subjected to 1% agarose gel electrophoresis and sequenced by Tsingke Biological Technology (Nanjing, China). Sequence alignments were performed using DNAMAN (version 6.0) (http://www.lynnon.com, accessed on 7 April 2022). The consensus sequence was aligned with available GenBank sequences, and a phylogenetic tree was constructed using MEGA 7.0 [31].

### 2.3. Quantification of Plant Hormones and Mineral Nutrients

Levels of the plant hormones, JA, SA, auxin, GA, CTK, ACC, BR, and ABA, were detected using high performance liquid chromatography-tandem mass spectrometry (HPLC-MS). In brief, each approximate 0.1 g sample was ground into a powder in liquid nitrogen and extracted with 7.9:2:0.1 methanol: water: formic acid containing sodium diethyldithiocarbamate at 4 °C. Then, the extracted solution was purified using a solid phase extraction process, including loading, washing, and eluting samples. The eluate solution was collected for the HPLC-MS (DGLC/Q exactive, Thermo Fisher Scientific, San Jose, CA, USA) analysis, according to the method of Fu et al. [32]. In addition, mineral nutrient contents were measured using the inductively coupled plasma mass spectrometry (ICP-MS) method. On the basis of the different mineral contents of samples, each approximate 0.01–0.05 g sample was extracted with nitric acid (approximately 1 mL) and shaken for 1 h in boiling water. Subsequently, the extracted solution was quantified using ICP-MS (PerkinElmer ELAN DRC-e, PerkinElmer ELAN Corporation, USA) [33]. The plant hormone contents and mineral data were assessed using SPSS 20.0 software to determine statistical significance. Duncan’s test at *p* = 0.05 was applied to identify significant differences among different groups.

### 2.4. RNA Extraction, cDNA Library Construction, and Sequencing

Total RNA was extracted from symptomatic and normal stems, with three biological replicates, using an RNAprep pure Plant Kit (TIANGEN, Beijing, China), following the manufacturer’s instructions. Then, the concentration and quality of total RNA were evaluated using a Nanodrop spectrophotometer. High-quality RNA samples were used for cDNA library construction. Briefly, the mRNA was isolated using oligo (dT) magnetic beads, and then fragments were randomly interrupted using fragmentation buffer. Using these short fragments, the double-stranded cDNA was synthesized with random hexamer primers and DNA polymerase I, followed by an RNase H treatment. The cDNA was purified, end-paired, then “A” bases were added, and adaptors ligated. It was then fragmented into approximately 200 bp pieces with AMPure XP beads for PCR applications. The six libraries were paired-end sequenced on an Illumina HiSeq 2500 system from Nanjing Genepioneer Biotechnologies Corporation (Nanjing, China). 

### 2.5. Bioinformatics Analysis of RNA-seq Data

The raw reads obtained from RNA-seq were processed by removing low-quality reads with Trimmomatic [34]. The Q20, Q30, and GC content of the total clean reads were then evaluated. Then, the high-quality clean reads were aligned to the cucumber Chinese long-draft genome assembly (http://cucurbitgenomics.org/organism/20, version 3, accessed on 11 October 2021) with HISAT2 [35]. The genes were sorted according to their fragments per kilobase per million (FPKM) value, which was used as an indicator for expression level. Differentially expressed genes (DEGs) between the symptomatic and healthy stems were identified by the R package, “DESeq”(version 1.12.1), with |log_2_(fold change)| ≥ 1 and false discovery rate < 0.01 [36,37]. These DEGs were mapped to the Gene Ontology (GO) database (http://www.geneontology.org/, accessed on 11 October 2021) and Kyoto Encyclopedia of Genes and Genomes (KEGG) database (http://www.genome.jp/kegg/, accessed on 11 October 2021) to analyze the function of DEGs. The GO and KEGG pathway enrichment analyses were carried out using topGO and clusterProfiler [38,39]. The *q-value* (a corrected *p*-value) < 0.01 was used as the cutoff for a significantly enriched GO term or KEGG pathway. The raw reads with available data were deposited in the sequence read archive of the National Center for Biotechnology Information, under accession number PRJNA731555.

### 2.6. qRT-PCR Analysis

To verify the reliability of the RNA-seq results, eight DEGs were selected to be assessed using qRT-PCR assays. The primers for the DEGs were designed using online Primer 3 software (https://primer3.ut.ee/, accessed on 23 October 2021), and the primers are listed in Table S1. qRT-PCR was performed with a SYBR Green mix (Vazyme, Nanjing, China) in accordance with the manufacturer’s instructions. The cucumber *β-actin* gene (*CsaV3_6G041900*) was used as a reference to normalize the transcription levels of each gene, with three biological replicates. The relative expression levels of the eight DEGs were calculated in accordance with the method of Livak and Schmittgen [40].

## 3. Results

### 3.1. Phytoplasma Infection Caused Flat Stem Symptoms in Cucumber

**Figure 1:**
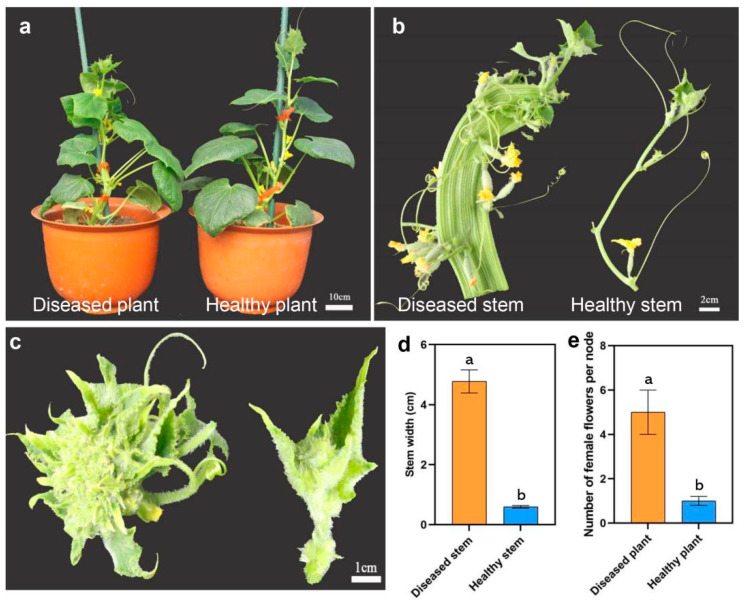
Diseased and healthy cucumber plants in spring 2021: (**a**) whole plants; (**b**) stems; (**c**) growth points; (**d**) stem width of the diseased and healthy cucumber plants; (**e**) number of female flowers of the diseased and healthy cucumber plants. Significance was determined by Duncan’s test (*p* < 0.05).

**Figure 2:**
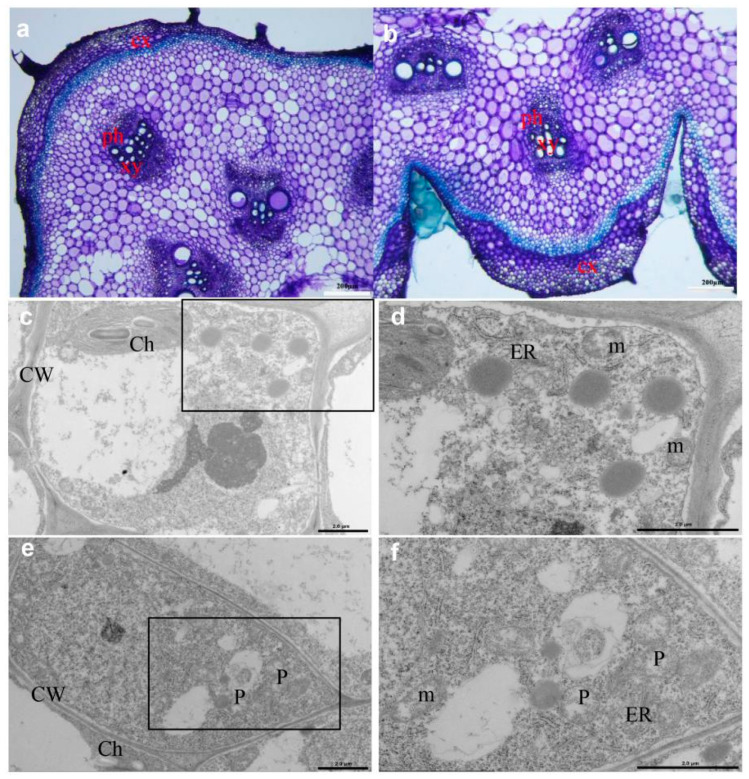
Microscopic images of stem sections in the diseased and healthy plants. Light microscopy graphs of healthy stems (**a**) and symptomatic stems (**b**). Transmission electron microscopy micrographs of stem sections in healthy plants (**c**,**d**) and diseased plants (**e**,**f**). CX: cortex; ph: phloem; xy: xylem; CW: cell wall; ch: chloroplast; ER: endoplasmic reticulum; m: mitochondrion; P: phytoplasma. Scale bars (**a**,**b**) = 200 μm; (**c**–**f**) = 2.0 μm. The black boxes show the locations of phytoplasmas.

The seedlings of the inbred C17 cucumber line were cultivated in a greenhouse under normal field management conditions at Yangzhou University in China. The flat stem and witches’ broom symptoms, which are typical of phytoplasma infections, were observed in diseased plants with three consecutive growing seasons (spring 2020, autumn 2020, and spring 2021), whereas the healthy plants did not have similar symptoms (Figure 1a–c). The diseased plants exhibited significant increases in stem width and female flower numbers than the healthy plants (Figure 1d,e). To explore the cause of flat stem formation, the stem transactions of healthy and diseased plants were compared (Figure 2). Compared with the healthy stems, the cortex thickness and vascular tissue thickness were increased in diseased stems (Figure 2a,b). The cellular ultrastructure changes were further examined by TEM. As expected, phytoplasmas were observed in the sieve tubes of flat stems but not in those of normal stems (Figure 2c–f). 

**Figure 3:**
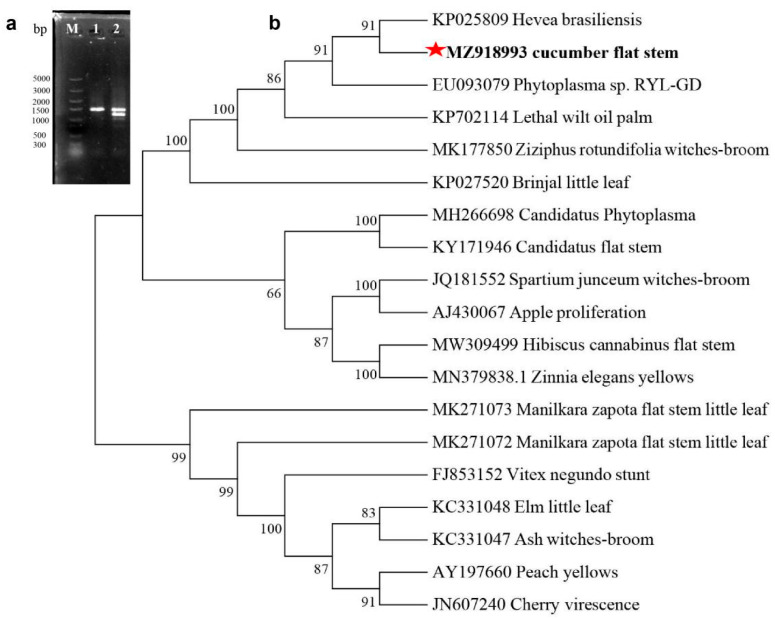
Detection and phylogenetic tree of 16S rRNA. (**a**) The amplification results of 16S rRNA by using nested PCR. M: Marker, lane 1 and 2 represents DNA extracted from the flat and healthy stems, respectively. (**b**) The phylogenetic tree of 16S rRNA gene sequences between the cucumber flat stem phytoplasma (the bold and red star) and several phytoplasma strains from GenBank. The bar indicates the phylogenetic distance based on the neighbor-joining method.

To further confirm the presence of phytoplasma, total DNA was independently extracted from the stem tissues of flat and normal stems. An approximate 1.23 kb fragment of 16S rRNA was amplified from symptomatic stems using nested PCR, with the primer pair P1/P7 followed by the primer pair R16F2n/R16R2, but no similar fragment was detected from normal stems (Figure 3a), suggesting the presence of cucumber flat stem phytoplasma. The sequence obtained from flat stems was submitted to GenBank under accession number MZ918993. A comparison of the full-length 16S rRNA sequence revealed an 84.43% identity with ‘*Hevea brasiliensis*’ phytoplasma (Acc. no. KP025809). To further classify the cucumber flat stem strain, a phylogenic tree was constructed using MEGA7.0 (Figure 3b). The cucumber flat stem strain clustered with ‘*Hevea brasiliensis*’ phytoplasma, lethal wilt oil palm phytoplasma, and ‘*Ziziphus rotundifolia*’ witches’ broom, which belongs to *Candidatus* phytoplasma. The ‘*Hevea brasiliensis*’ phytoplasma has been detected in rubber trees and produces stem fasciation and witches’ broom symptoms [41]. These results suggested that the phytoplasma from the flat stems of cucumber was a *Candidatus* phytoplasma *asteris* strain.

### 3.2. Phytoplasma Infection Caused Cucumber Plant Hormone Disorders

**Figure 4:**
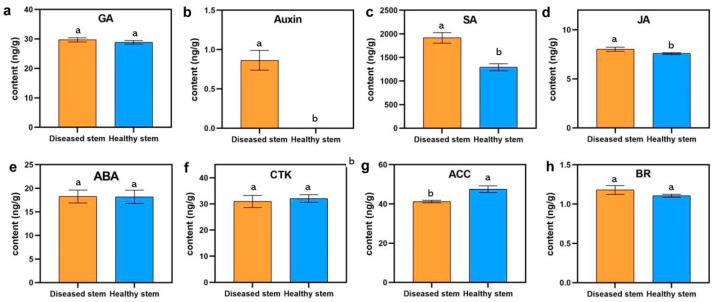
Endogenous plant hormone contents in flat and healthy cucumber stems: (**a**) gibberellin (GA); (**b**) auxin; (**c**) salicylic acid (SA); (**d**) jasmonic acid (JA); (**e**) abscisic acid (ABA); (**f**) cytokinin (CTK); (**g**) 1-aminocyclopropane-1-carboxylic acid (ACC); and (**h**) brassinolide (BR). Different letters represent significant differences in different groups (*p* < 0.05, Duncan’ test).

To explore the changes in plant hormones during phytoplasma infections in cucumber, the contents of eight plant hormones were measured in flat and normal stems using high-performance liquid chromatography-mass spectrometry (HPLC-MS) (Figure 4). Four of the tested plant hormones showed no significant differences between symptomatic and normal stems, including GA, ABA, CTK, and BR (Figure 4a,e,f,h). Interestingly, auxin was not detected in healthy stems, but was significantly increased in diseased stems, indicating that auxin has a crucial influence on responses to pathogen attacks (Figure 4b). In addition, the pathogen infection of cucumber resulted in SA and JA contents significantly increasing, whereas ACC content significantly decreased (Figure 4c,d,g). These results indicated that SA, JA, and ETH regulate the defenses of cucumber against invading pathogens.

### 3.3. Phytoplasma Infection Caused Mineral Element Disorders

**Figure 5:**
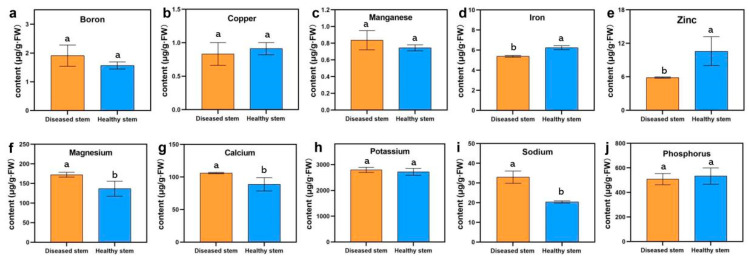
Mineral contents in flat and healthy cucumber stems: (**a**) boron; (**b**) copper; (**c**) manganese; (**d**) iron; (**e**) zinc; (**f**) magnesium; (**g**) calcium; (**h**) potassium; (**i**) sodium; and (**j**) phosphorus. Different letters represent significant differences between flat and healthy stems using Duncan’s test (*p* < 0.05).

Mineral nutrients are necessary to maintain plant growth and development, and as the phytoplasma infection could affect the absorption and transformation of cucumber, the concentrations of nine mineral elements for plant growth and sodium of symptomatic and healthy stems were analyzed using inductively coupled plasma-mass spectrometry (ICP-MS) (Figure 5). The results suggested that iron, zinc, copper, manganese, and boron contents were low in cucumber stems (Figure 5a–e) and that iron and zinc concentrations in healthy stems were significantly higher than in flat stems. However, manganese and boron concentrations were reduced in healthy stems. In addition, calcium, magnesium, and sodium were significantly accumulated in the symptomatic plants compared with healthy plants (Figure 5f,g,i), suggesting that these mineral elements enhance the ability of cucumber to defend against the microbes.

### 3.4. Phytoplasma Infection Influences Gene Expression

To explore the molecular mechanisms behind the formation of flat stems, we performed an RNA-seq analysis using the flat and normal stems from the C17 cucumber inbred line. High-throughput RNA-seq resulted in 40.17 to 49.94 million paired-end reads for each sample, with three repetitions (Table S2). After removing low-quality reads and adapter sequences, 38.96 to 48.12 million clean reads were obtained, among which 89.98 to 92.65% of reads were uniquely mapped to the reference genome. The Q30 percentage and GC content were greater than 92.50 and 42.85%, respectively. 

**Figure 6:**
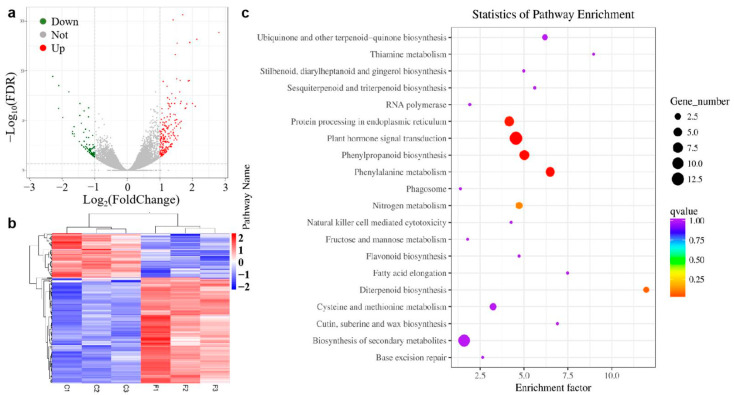
RNA-seq analysis. (**a**) Visualization of RNA-seq results with a volcano plot. The red dots represent differentially expressed genes (DEGs) with log_2_(fold change) ≥ 1 and false discovery rates (FDR) < 0.01, whereas green dots represent DEGs with log_2_(fold change) ≤ −1 and FDR < 0.01; the black dots represent insignificant DEGs with FC between 0.5 and 2 or FDR ≥ 0.01. (**b**) Hierarchical clustering of analyses of DEGs in the stems of normal (C) and symptomatic (F) plants; 1, 2, and 3 represent the three repetitions. (**c**) Kyoto Encyclopedia of Genes and Genomes (KEGG) pathway enrichment analysis of the DEGs. The X-axis represents the enrichment factor, and Y-axis represents the KEGG pathway. The size of each dot indicates the gene number that was annotated in this pathway. The dot color represents the *q-value*, with red dots indicating that the pathway was significantly enriched.

Pairwise comparisons of genes having false discovery rates (FDR) < 0.01 and |log_2_(fold change)| ≥ 1 in abundance were regarded as differentially expressed genes (DEGs). We identified 253 DEGs, including 179 upregulated and 74 downregulated genes, in a comparison between flat and healthy stems (Figure 6a, details in Table S3). Hierarchical clustering analysis using the FPKM values of DEGs revealed that the biological replicates were grouped together, indicating high reliability of the generated RNA-seq data (Figure 6b). To further confirm the reliability of the RNA-seq data, we randomly selected eight DEGs to assess using qRT-PCR assays. As shown in Figure S1, there was a strong positive correlation (two tailed, R^2^ = 0.84) between the RNA-seq data and qRT-PCR data, further indicating the credibility of the RNA-seq data.

To identify the potential biological functions of the DEGs, a Kyoto Encyclopedia of Genes and Genomes (KEGG) pathway enrichment analysis was performed (Table S4). DEGs were mapped to 32 KEGG pathways and the top 20 KEGG pathways are shown in Figure 6c. We found that four pathways were significantly enriched (*q-value* < 0.1), which included plant hormone signal transduction (ko04075, 11 DEGs), phenylalanine metabolism (ko00360, 6 DEGs), phenylpropanoid biosynthesis (ko00940, 7 DEGs), protein processing in endoplasmic reticulum (ko04141, 7 DEGs). In addition, gene ontology (GO) enrichment was further performed with *q-value* < 0.01 as the threshold value, and nine significantly enriched GO terms were identified, including auxin-activated signaling pathway (GO: 0009734), auxin biosynthesis process (GO: 0009851), L-phenylalanine catabolic process (GO: 0006559), and phenylalanine ammonia-lyase activity (GO: 0045548) (Table S5).

### 3.5. DEGs Related to Plant Hormone Signal Transduction

**Figure 7:**
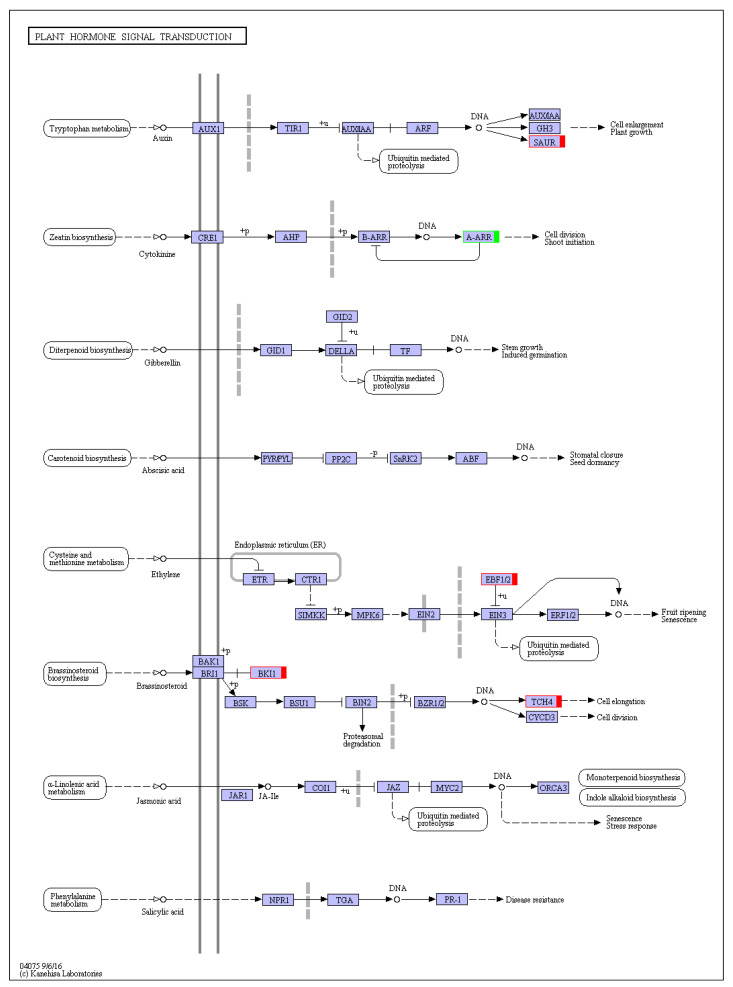
Differentially expressed genes (DEGs) mapped to the plant hormone signal transduction pathway. The red and green box represent upregulated and downregulated encoding genes in the flat stems compared with healthy stems, respectively.

The KEGG pathway enrichment analyses revealed that the plant hormone signal transduction pathway was the most significantly enriched pathway. A total of 11 DEGs involved in plant hormone and signal transduction were identified (Figure 7, details in Table S6). In total, seven DEGs were related to auxin, including *CsaV3_2G015500*, *CsaV3_2G015510*, *CsaV3_2G015520*, *CsaV3_2G015550*, and *CsaV3_2G015560*, which encodes small auxin up RNA (SAUR) and *CsaV3_7G006000* and *CsaV3_7G00620*, encoding auxin-induced proteins. These DEGs involved in the auxin active signal pathway were upregulated in flat stems, which may be due to increased auxin content in flat stems **(**Figure 4b), indicating that auxin plays an important role in regulating cucumber–pathogen interactions. In addition, a CTK downstream response regulator, *CsARR17* (*CsaV3_6G034340*), had low transcription levels in symptomatic stems resulted in the initiation of CTK synthesis [42]. *CsBKI1* (*CsaV3_2G027990*), which is related to BR signal transduction, and xyloglucan endotransglucosylase/hydrolase (*CsaV3_1G031430*), which is involved in BR-activated cell elongation, was upregulated in flat stems and resulted in enhanced BR content (Figure 4h). *CsaV3_3G046430*, encoding the EIN3-binding F-box protein, which negatively regulates ETH signal transduction, was upregulated in flat stems, indicating ETH may be decreased in the flat stems, which was consistent with the determination of ACC content (Figure 4g). In conclusion, auxin, CTK, BR, and ETH were important mediators in the cucumber responses against the pathogen.

### 3.6. Identification of DEGs Related to Secondary Metabolite Biosynthesis

**Figure 8:**
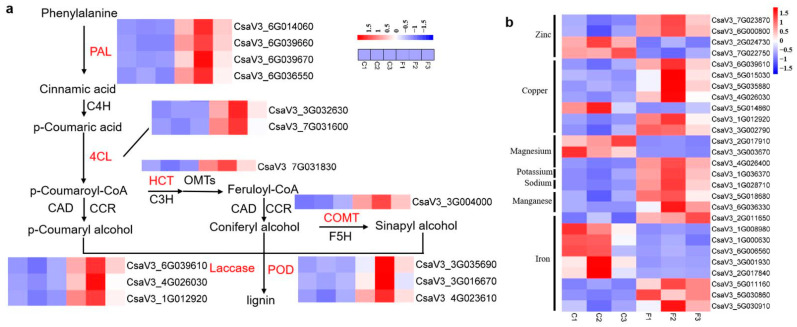
Heatmap of differentially expressed genes (DEGs) related to plant disease resistance in the stems of normal (C) and symptomatic (F) plants; 1, 2, and 3 represent the three repetitions. (**a**) DEGs involved in phenylalanine biosynthetic and lignin biosynthetic pathways. (**b**) DEGs related to mineral ions. A red rectangle indicates that the transcription level of the gene was upregulated, and a blue rectangle indicates that the transcription level of the gene was downregulated.

Secondary metabolites, such as phenylpropanoid, play important roles in plant responses to both abiotic and biotic stresses [43]. The phenylpropanoid biosynthesis pathway begins with phenylalanine, which can be converted into aromatic compounds, including benzenoids, coumarins, flavonoids, and lignin. In this study, phenylalanine metabolism and phenylpropanoid biosynthesis were significantly enriched pathways in the flat stem versus healthy stem comparison. A total of twelve DEGs were identified, including four genes encoding phenylalanine ammonia-lyase (PAL), two genes encoding 4 coumarate COA ligase (4CL), *CsaV3_7G031830*, encoding hydroxycinnamoyl-CoA shikimate, *CsaV3_3G00400* encoding caffeic acid 3-O-methyltransferase, three genes encoding laccase, and two genes encoding POD (Figure 8a). The transcription levels of the 14 genes were increased in symptomatic plants, indicating that these plants may use enhanced lignin levels to fight against pathogen infections.

### 3.7. DEGs Involved in Mineral Nutrition Resist Phytoplasma Infection

To investigate the effect of phytoplasma on mineral nutrients in the infected cucumber, we analyzed the expression levels of mineral ion-related DEGs based on gene ontology (GO) annotation (Table S3). There were 11 GO terms, zinc ion binding (GO: 0008270), copper ion binding (GO: 0005507), iron ion binding (GO: 0005506), manganese ion binding (GO: 0030145), magnesium ion binding (GO: 0000287), response to zinc ion (GO: 0010043), copper ion transport (GO: 0006825), cellular copper ion homeostasis (GO: 0006878), transition metal ion binding (GO: 0046914), potassium ion transmembrane transporter activity (GO: 0015079), and cellular sodium ion homeostasis (GO: 0006883), with 27 DEGs related to mineral elements in the flat stem versus healthy stem. As shown in Figure 8b, *CsaV3_1G028710*, related to cell sodium ions, was induced in the symptomatic stem, suggesting that sodium content could be increased in the flat stem **(**Figure 5i). Germin-like protein members, *CsaV3_5G018680* and *CsaV3_6G036330*, involved in manganese ion binding, were upregulated in the flat stem, which resulted in increased manganese content (Figure 5c). *CsaV3_1G036370* encoding the potassium ion transporter was downregulated in the healthy stem, causing variation in potassium content; however, the change was not obvious (Figure 5h). In addition, 20 DEGs related to zinc, copper, and iron ion binding were induced or inhibited, which resulted in the alteration of copper, iron, and zinc concentrations in the cucumber stem after being infected by phytoplasma (Figure 5b,d,e).

## 4. Discussion

### 4.1. Identification of the Cucumber Flat Stem Pathogen

Diseases caused by phytoplasmas have been reported in *Celosia argentea* L., *Impatiens balsamina*, and spinach [44,45,46]. Phytoplasmas are obligate parasites that present in the phloem sieve tube cells of plants. They are transmitted by insects, especially leafhoppers and planthoppers [5]. In cucumber, phyllody caused by phytoplasma has only ever been reported in Iran [26]. In the present study, we demonstrated that cucumber was infected with phytoplasma using light microscopy, TEM, nested PCR, and sequencing. The phytoplasma infection resulted in the formation of flat stems and witches’ broom, and the number of female flowers significantly increased. This is the first report of cucumber infected with phytoplasma in China, and further research on phytoplasma infection mechanisms in cucumber is needed.

### 4.2. Alterations in Plant Hormone Levels in Response to Phytoplasma Infection

Recent studies have revealed an important role for plant hormones in plant–pathogen interactions [47]. In our study, the plant hormone contents dramatically changed, and a number of DEGs involved in plant hormone biosynthesis and signal transduction were identified during the phytoplasma infection process, including auxin, CTK, BR, SA, JA, and ETH. Our results further confirmed that plant hormones play crucial roles in the defense of cucumber against phytoplasma. 

In periwinkle, an auxin imbalance is a critical factor in phytoplasma-related symptom development [48]. In the early stages of witches’ broom (JWB) phytoplasma infection of Chinese jujube, symptoms are invisible, but genes involved in auxin biosynthesis and signal transduction are repressed, and corresponding auxin contents were decreased in infected leaves. However, the expression levels of these genes were not significantly different during the later stages of JWB infection when significant witches’ broom symptoms appeared [8]. In our study, five DEGs encoding SAUR family proteins, the early stages of the auxin-response gene [49], were induced in infected stems, which was consistent with Mardi’s research that SAURs are induced in the leaves of Mexican lime trees infected with ‘*Ca.* P. aurantifolia’ phytoplasma [50]. Moreover, in coconut, genes encoding auxin-induced proteins were overexpressed during coconut yellow decline (CYD) phytoplasma infection [16]. Similar results were found in cucumber during phytoplasma infection. The upregulated auxin response or induced genes in the flat stems may be due to an increase in auxin content compared with healthy plants. Thus, auxin may be directly involved in defending against pathogen invasion.

The involvement of CTK in plant defenses against phytoplasma infection has been reported in other plants, such as mulberry [51], coconut [16], and paulownia [52]. The transcription level of the gene encoding cytokinin dehydrogenase was lower in leaf midribs of coconut plants infected with CYD phytoplasma compared with leaf midribs of uninfected plants. Similarly, *CsaV3_6G034340* that encoded the CTK downstream response regulator *ARR17*, was downregulated in the infected stems [53]. Accordingly, the CTK content showed slightly decreased levels in the flat stem, suggesting that CTK was associated with defending against pathogen infection in cucumber.

In addition, genes related to BR biosynthesis and signal transduction were reported to be upregulated in response to plant–pathogen interactions [17,54,55]. Here, we found that the gene encoding BRI1 kinase inhibitor, a positive regulator of the BR signal [56], was upregulated in flat stems, indicating that BR signals may be involved in the cucumber defense against pathogen attack. 

In plant immunity, SA, JA, and ETH act as the main hormones [57]. Some genes involved in SA biosynthesis and signal transduction, such as PAL and nonexpressor of pathogenesis-related genes, were upregulated in the leaves of grapevines infected with ‘*Candidatus* phytoplasma solani’ phytoplasma, which resulted in significantly increased SA levels [58]. We found the *CsaV3_6G042950* encoding SA-binding protein 2 was upregulated in infected cucumber stems. Overexpression of the *LcSABP2*, a salicylic acid binding protein 2 gene from *Lycium chinense*, resulted in enhanced SA levels [59]. The SA content was significantly increased in this study, suggesting that SA plays a crucial role in cucumber responses to pathogen infection. 

Genes related to ETH biosynthesis and signaling were significantly differentially expressed in infected plants [50]. These genes included ACC oxidase, ACC synthase, AP2/ERF, and ETH-responsive transduction factors. For example, Fan et al. reported that DEGs related to ACC oxidase and ACC synthase were significantly induced in paulownia infected with PWB phytoplasma [6]. Our analysis implied that the gene that encodes ACC oxidase (*CsaV3_6G015400*), which catalyzes the synthesis of ethylene from ACC [60], was upregulated in infected cucumber, but the gene encoding ACC synthase (*CsaV3_2G025850*) that catalyzes the synthesis of ACC was repressed [60], which resulted in decreased ACC contents in the flat stems. These results were in agreement with the research of Broekaert et al. [61], in which ACC oxidase and ACC synthase had different expression patterns in response to pathogen infection, suggesting that ETH has an important function in cucumber defense against pathogen infection.

Several genes related to JA biosynthesis and signal transduction were induced during JWB phytoplasma infection, such as lipoxygenase 2 and JA-induced protein; the JA levels increased during the early stage of JWB phytoplasma infection, suggesting that JA contributes to defenses against pathogen infection [8]. Our RNA-seq analysis revealed that a DEG (*CsaV3_1G000630*) encoding cytochrome P450, which regulates the catabolism of jasmonoyl-L-isoleucine, was downregulated in flat stems [62]. This may have resulted in the increase in jasmonoyl-L-isoleucine content (Figure 4d), indicating that JA also plays a crucial role during cucumber defense against invading phytoplasma.

### 4.3. Phenylpropanoid Genes Related to Plant Disease Resistance

Both PAL and 4CL are enzymes involved in phenylpropanoid biosynthesis, and they are upregulated in jujube infected with phytoplasma [6,10]. Here, six DEGs encoding PAL and 4CL were induced in infected cucumber, suggesting that phenylpropanoid plays an important role during phytoplasma attack. Fan et al. found that the expression levels of genes encoding shikimate O-hydroxycinnamoyltransferase, cinnamoyl CoA reductase, cinnamyl alcohol dehydrogenase, caffeic acid 3-O-methyltransferase, and POD in infected paulownia were higher than in healthy plants, and this contributed to the reinforcement of the cell wall to defend against PWB phytoplasma attack [6]. Similarly, in our study, eight genes involved in the lignin biosynthetic pathway were upregulated in response to cucumber flat stem phytoplasma infection (Figure 8a). Thus, we hypothesize that cucumber uses lignification to enhance its defenses against phytoplasmas.

### 4.4. Mineral Nutrient Regulation of Plant-Pathogen Interactions

In plants, mineral nutrients play important roles in responding to pathogen infection [21]. They change the growth manner and morphological structures of plants. For example, they thicken cell walls, increase lignification and silicification to form a mechanical barrier, and produce many materials that enhance plant resistance [63]. Bakeer et al. reported that increases in iron and zinc contents could lessen the severity of powdery mildew in tomato [64]. In citrus, iron and zinc concentrations are low in leaves after citrus is infected with *Candidatus* Liberibacter asiaticus [65]. In this study, iron and zinc contents were low in infected stems, suggesting that the lack of iron and zinc increased disease severity. Treatment with a high calcium nutrient solution decreased disease severity in tomato [66]. Tomato absorbed more calcium, thereby enhancing the activities of H_2_O_2_ and POD, which increased disease resistance [65]. However, calcium and magnesium concentrations were decreased in the midrib and lamina of phytoplasma-infected tomato, respectively [67]. Calcium and boron concentrations were reduced in tomato leaves inoculated with the bacterial wilt causal agent [68]. On the contrary, our analysis indicated that calcium and magnesium content significantly increased, and boron content was reduced in symptomatic plants compared with healthy plants, which may be that the stems were responsible for the transportation of mineral nutrition and leaves for energy capture [69]. Moreover, when citrus was infected with phytoplasma, sodium, phosphorus, and potassium contents were significantly induced [70]. Raiesi and Golmohammadi [71] reported that phosphorus, and potassium contents increased in infected leaves and roots. Similarly, the sodium contents were increased in infected cucumber stems, whereas the phosphorus and potassium contents showed no difference. However, the potassium transporter protein (*CsaV3_1G036370*) was induced in the flat stems. As expected, an imbalance of the potassium/sodium ratio in the symptomatic stems could affect cellular ion homeostasis [67].

## 5. Conclusions

In summary, due to phytoplasma infection, cucumber exhibited flat stems and witches’ broom symptoms. Further mineral element and plant hormone content measurement and RNA-seq analysis revealed that auxin, SA, ETH, JA, and some mineral nutriments, such as magnesium, calcium, sodium, iron, zinc, and phenylpropanoid, play crucial roles in defense against pathogen invasion, which increased our understanding of the underlying responses of cucumber to phytoplasma infection.

## Data Availability

The 16S rRNA sequence of cucumber was deposited in GenBank, under accession number MZ918993. The raw sequence data of this study were deposited in the sequence read archive of the National Center for Biotechnology Information, under accession number PRJNA731555.

## Supplementary Materials

The following supporting information can be downloaded at: https://www.mdpi.com/article/10.3390/genes13101903/s1, Table S1: Information regarding the primers used in this study; Table S2: Summary of RNA-seq data statistics; Table S3: List of differentially expressed genes identified in this study; Table S4: KEGG pathway enrichment analysis of differentially expressed genes identified in this study; Table S5: GO enrichment analysis of differentially expressed genes identified in this study; Table S6: List of differentially expressed genes involved plant hormone signal transduction pathway; Figure S1: Correlation analysis of the relative expression levels of eight randomly selected differently expressed genes between the RNA-seq and qPCR.
