# Supplementary figures and images for: Comparative Biochemical and Transcriptomic Analyses Provide New Insights into Phytoplasma Infection Responses in Cucumber
Source: Genes (Basel). 2022 Oct 19;13(10):1903. doi: 10.3390/genes13101903 (PMC9602156; doi:10.3390/genes13101903)

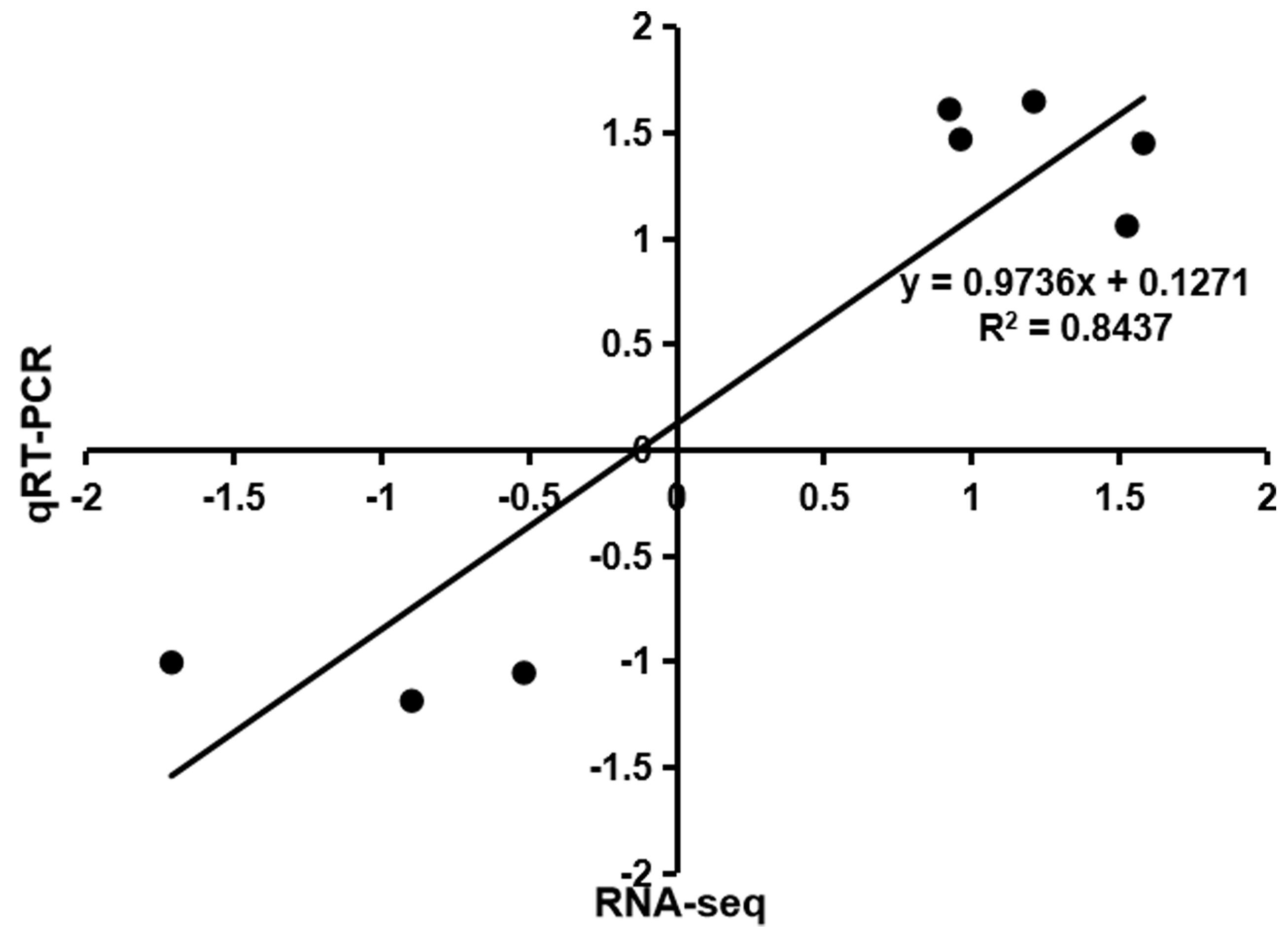

Supplement: Supplementary file 1 [file genes-13-01903-s001.zip › FigureS1.jpg]
